# Extrinsic and Cell-Intrinsic Stress in the Immune Tumor Micro-Environment

**DOI:** 10.3390/ijms252212403

**Published:** 2024-11-19

**Authors:** Aldo Ummarino, Nicholas Calà, Paola Allavena

**Affiliations:** 1 Department of Biomedical Sciences, Humanitas University, 20072 Milan, Italy; aldo.ummarino@childrens.harvard.edu; 2 IRCCS Humanitas Research Hospital, 20089 Milan, Italy; 3 Etromapmacs Pole, Agorà Biomedical Sciences, 71010 Foggia, Italy; ncala@scienzebiomediche.it

**Keywords:** metabolism, hypoxia, tumor-associated macrophages, tumor micro-environment, cancer-related inflammation

## Abstract

In continuously progressive tumor tissues, the causes of cellular stress are multiple: metabolic alterations, nutrient deprivation, chronic inflammation and hypoxia. To survive, tumor cells activate the stress response program, a highly conserved molecular reprogramming proposed to cope with challenges in a hostile environment. Not only cancer cells are affected, but stress responses in tumors also have a profound impact on their normal cellular counterparts: fibroblasts, endothelial cells and infiltrating immune cells. In recent years, there has been a growing interest in the interaction between cancer and immune cells, especially in difficult conditions of cellular stress. A growing literature indicates that knowledge of the molecular pathways activated in tumor and immune cells under stress conditions may offer new insights for possible therapeutic interventions. Counter-regulating the stress caused by the presence of a growing tumor can therefore be a weapon to limit disease progression. Here, we review the main pathways activated in cellular stress responses with a focus on immune cells present in the tumor microenvironment.

## 1. Introduction

Tissue homeostasis is characterized by a steady state without inflammation or other potential harmful reactions, adequate blood circulation with constant oxygen supply, and balanced metabolic turnover. The presence of a growing tumor causes a profound alteration of the tissue because tumors have a greater demand for nutrients due to the high proliferation rate, increased oxygen consumption and altered metabolism. 

The tumor microenvironment (TME) is made up of a heterogeneous set of different cell types, among which, in addition to malignant cells, there are cells of the immune system, vascular and lymphatic endothelial cells and fibroblasts. In this complex ecosystem, immune cells are mainly represented by different subsets of myeloid and lymphoid cells that derive from blood-circulating precursors, although some tissue-resident cells, notably macrophages, also populate the tumor stroma [1,2,3,4,5,6,7,8,9].

A distinctive feature of tumor cells is that they implement specific conditioning to their advantage. For example, they produce pro-angiogenic factors to stimulate the vascular network and receive more blood-derived nutrients and oxygen; they activate fibroblasts to remodel the extracellular matrix and produce inhibitory factors that suppress anti-tumor immune responses; they secrete a plethora of chemokines inducing the recruitment of myeloid immunosuppressive cells. However, the presence of neoplastic cells can be perceived as “danger” by the innate immunity, and tumor-associated antigens can stimulate adaptive T cells to react against them. Therefore, the interaction of immune cells with neoplastic cells and which equilibrium will predominate over time is of crucial importance for the balance between tumor survival and tumor elimination by an active anti-tumor immune response [1,2,3,4,5,6,7,8].

In recent decades, there has been extraordinary interest in immune cells that infiltrate tumors. Several studies on the composition, phenotype and functional characteristics of tumor-infiltrating leukocytes have been published, especially boosted by the advanced single-cell and spatial transcriptomic technologies [10,11,12,13,14].

A much more complex picture emerged than previously detected. Tumor-Associated Macrophages (TAMs) constitute the most abundant immune population in solid tumors. TAMs are very heterogeneous and can be classified into several different clusters with specific genetic markers and predicted diverse functional activities. The initial idea about mononuclear phagocyte heterogeneity (a concept known since the 90s) was based on a simple binary division: M1 (or classically activated) macrophages stimulated by bacterial components (e.g., LPS or other TLR ligands) and cytokines such as IFNs have inflammatory immunostimulatory functions and anti-tumor activity, while M2 (or alternatively activated) macrophages stimulated by cytokines such as IL-4 and IL-10, have immunosuppressive, anti-inflammatory functions and favor tumor progression. This M1/M2 dichotomy proved to be no longer suitable for TAMs on the basis of new knowledge acquired [1,15,16,17,18,19,20]. However, it remains confirmed that in the vast majority of human tumors and especially in advanced tumors, TAMs promote a tumor-supportive and immunosuppressive environment, and their abundance in tumors is associated with poor patient prognosis. TAMs support tumor development in many different ways. They actively promote cancer cell division, invasion and metastasis formation, angiogenesis, matrix deposition and degradation and repression of adaptive immunity [4,5,6,7,8].

This knowledge has led to the development of a series of studies to target macrophages in tumors with the aim of reducing their number or reprogramming their functional activity in an anti-tumor mode. Different strategies have been pursued, for example, using compounds or mAbs that inhibit receptors or immunosuppressive pathways or, vice versa, that activate potential stimulatory functions. A large number of reviews have been published on this topic [4,6,7,9,21,22,23].

The tumor environment is a place of stress. Neoplastic cells face internal and environmental challenges such as genomic instability, continuous replication, hypoxia and nutrient deprivation. In these unfavorable situations, they activate specific stress response programs that allow them to avoid cellular dysfunction and death. Stress-evoked protective responses include increased protein synthesis and folding, an increased rate of metabolism and activation of autophagy to remove damaged organelles and exploit residual nutrients [24,25,26]. 

**Figure 1:**
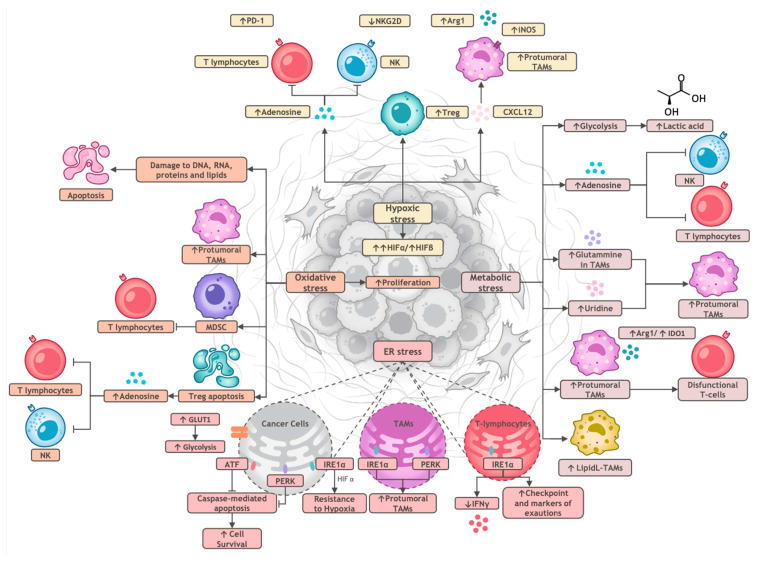
Principal determinants of cellular stress in the TME. Because of their proliferation, cancer cells are exposed to four types of stress: hypoxic, metabolic, ER and oxidative. Hypoxic stress (upper part of the figure) induces immunosuppressive T regulatory (Treg) cells, as well as the release of adenosine from cancer cells, inducing PD1 on lymphocytes and reducing the expression of the activating receptor NKG2D on NK cells. In addition, cancer cells in the hypoxic TME produce CXCL12 that recruits protumoral TAMs. Metabolic stress (right part of the figure) induces the release of lactic acid, altering the pH of the TME, as well as of glutamine and uridine, affecting TAMs. ER stress (lower part of the figure) in cancer cells prevents apoptosis through ATF and PERK as well as induces resistance to hypoxia through IRE1α and HIFα. IRE1α induces also protumoral TAMs as well as the reduction in the release of IFNγ and the expression of checkpoint markers from T cells. Lastly, oxidative stress (left part of the figure) damages directly DNA, RNA and other molecules, leading to apoptosis in different cell types. The death of Treg leads to the release of adenosine, suppressive on T and NK cells. Lastly, oxidative stress induces MDSC, with suppressive activity on intratumoral T lymphocytes. Arg1 = Arginase 1; ATF = Activating transcription factor; GLUT1 = Glucose transporter 1; HIFα = Hypoxia-inducible factor 1-alpha; IDO1 = Indoleamine 2,3-dioxygenase; iNOS = inducible nitric oxide synthase; IRE1α = inositol-requiring enzyme type 1α; MDSC = myeloid-derived suppressor cells; NK = Natural Killer cells; PERK = protein kinase R-like endoplasmic reticulum kinase; TAMs = Tumor-Associated Macrophages.

Stress responses within the tumor microenvironment have a significant impact on the immune cells that populate the tumor; in particular, innate immunity cells such as monocytes-macrophages sense these environmental changes as a danger and respond by activating functional pathways that paradoxically can also be beneficial for the tumor. Here, we explore how typical cellular stress conditions within the tumor microenvironment affect infiltrating immune cells and discuss the potential of targeting specific pathways for therapeutic interventions. The main sources of stress in the TME are summarized in Figure 1 and discussed in the next paragraphs.

## 2. Hypoxic Stress

The availability of oxygen is essential for cellular metabolism in aerobic organisms; a low concentration of oxygen in tissues, a condition called “hypoxia”, leads to cellular stress. Several pathological conditions are characterized by low oxygen tension in tissues: chronic inflammation, ischemia, atherosclerosis and tumors [27,28].

Oxygen-sensing mechanisms within the cells trigger specific transcriptional responses to activate the transcription of genes that mediate adaptation to low oxygen conditions [29,30,31]. 

In solid tumors characterized by rapid growth and dysfunctional blood vessel network, hypoxia is almost invariably present. Hypoxia-inducible factors (HIFs) are the main transcriptional activators and regulate genes linked to crucial biological processes, including angiogenesis, cell proliferation and migration, glucose and lipid metabolism. Therefore, activation of HIF signaling in tumors strongly supports cancer growth and invasion. 

Hypoxia-induced tumor progression is also mediated through several types of immune cells that are modulated to become immunosuppressive or exhausted effectors. For example, hypoxia increases the generation of adenosine from cancer cells, which inhibits the cytotoxic activity of T lymphocytes and NK cells by triggering intracellular cyclic AMP production [32]. Their tumor-killing ability is hampered also because hypoxia downregulates the expression of the NKG2D activating receptor on NK cells and upregulates the inhibitory receptors PD-1 and LAG-3 [33,34]. In addition, hypoxic conditions stimulate the accumulation of suppressive Treg cells, abrogating the activity of potential anti-tumor responses [35]. 

Myeloid cells in the TME are especially affected by hypoxic conditions. Low oxygen tension induces the production of pro-angiogenic factors in an attempt to re-establish an adequate blood supply. In hypoxic tumor areas, VEGF and the CXCL12-CXCR4 chemotactic axis stimulate monocyte/macrophage recruitment. Therefore, TAMs are particularly abundant in hypoxic areas [36].

Two isoforms of the oxygen sensor HIF, namely HIF-1α and HIF2-α, show some opposite effects: HIF-1α is dominant in the acute phase of hypoxia and leads to gene transcription of inflammatory cytokines and inducible nitric oxide synthase (iNOS), typical of a proinflammatory phenotype. Instead, in chronic hypoxia, HIF-2α predominates, resulting in increased arginase 1 expression, suppression of iNOS, and a shift to an immune-tolerant phenotype. Hypoxia and low nutrient availability also strongly influence the metabolic reprogramming of TAMs towards a glycolytic mode, again shifting towards an immunosuppressive phenotype. Protumoral TAMs provide trophic functions for tumor cells, and their abundance in tumors is associated with an unfavorable prognosis and a reduced response to therapies [7,26,37].

## 3. Oxidative Stress

Cellular aerobic metabolism produces oxygen-derived byproducts, commonly known as reactive oxygen (ROS) or nitrogen (RNS) species. ROS are highly reactive chemicals; under normal circumstances, the cellular redox state is maintained by balancing the production of antioxidant molecules that capture and neutralize oxygen free radicals [38,39].

An excessive increase in ROS causes a pathological process, resulting in oxidative damage to many cellular molecules: RNA, DNA, proteins and lipids, ultimately triggering cell apoptosis. Excessive ROS has been reported to be tightly linked with events that characterize several pathological conditions, such as chronic inflammation, neurodegenerative and cardiovascular diseases, aging and cancer.

In the tumor tissue, where the metabolic demand is constantly increasing, cancer cells produce high ROS levels. ROS function as secondary messengers that activate transcription factors involved in cellular adaptation to stress and regulation of immunity. Several studies have documented a connection between cell proliferation and ROS, since these metabolites, and especially hydrogen peroxide (H_2_O_2_), can inactivate phosphatases that negatively regulate the proliferative pathways. Together with increased proliferation, ROS induce DNA damage, thus promoting tumorigenesis, the escape of tumor cells from apoptosis and the invasion of surrounding tissues [40,41]. Additionally, reactive oxygen and nitrogen species have been implicated in the oxidative stress that affects the TME and impacts on the infiltrating immune cells, primarily on the immunosuppressive myeloid-derived suppressor cells (MDSCs) and TAMs [42,43]. 

In lung and breast mouse cancer models, TAMs require ROS to infiltrate the tumor niche and to differentiate into a protumorigenic phenotype. Suppression of T cells by MDSC also require ROS, and neutralization of H_2_O_2_ reactivity by the enzyme catalase restores IFNγ production in suppressed T cells [44,45].

Nuclear factor erythroid 2-related factor 2 (NFE2L2), also known as NRF2, is a key transcription factor that controls the expression of various antioxidant enzymes. The regulation of NRF2 is particularly complex; studies have shown that its activation can counteract tumor transformation and early growth by effectively reducing oxidative stress; however, its prolonged constitutive activation may contribute to tumor progression and drug resistance. The paradoxical tumor-suppressing and tumor-promoting effects of NRF2 may depend on its protein levels, duration of activation, or crosstalk with specific factors. NRF2 expression is often elevated in tumor cells. Less is known about NRF2 activation and its consequences in immune cells present in the tumor microenvironment. It should be useful to remember that during homeostasis, ROS produced by phagocytes is a crucial mechanism for clearing bacterial and viral infections. Different results are observed in the stressed TME. Recent studies have reported that NRF2 causes transcriptional and metabolic reprogramming in myeloid cells; high levels of NRF2 in TAMs inhibit their potential cytotoxicity on tumor cells and also impair T cell-mediated antitumor responses [46,47]. Regulatory T cells are poor in antioxidant molecules such as NRF2 and particularly vulnerable to oxidative stress. However, apoptotic T regs maintain their suppressive capacity by releasing adenosine, which dampens the antitumor activity of T and NK cells and also promotes TAM and MDSC immunosuppressive activity. Overall, the oxidative stress affects the whole TME and results in severely compromised immune functions, tumor evasion from the immune system, disease progression and therapy resistance.

## 4. Metabolic Stress

The tumor tissue is characterized by a profound change in cellular metabolism caused by nutrient deprivation and hypoxia. As first described in the 1930s, tumor cells produce energy not through the usual oxidative phosphorylation in the mitochondria but through anaerobic glycolysis that produces lactic acid (the Warburg effect). Metabolic reprogramming from oxidative phosphorylation to glycolysis is a hallmark of cancer and ultimately a huge advantage for tumor cell growth. The normal counterpart of the tumor stroma, in particular the immune compartment (T lymphocytes and NK cells, macrophages and dendritic cells), also undergoes metabolic rewiring with a dramatic impact on their effector function. For mononuclear phagocytes in particular, metabolic changes in response to signals from the TME have emerged as major modulators of their functional polarization, most frequently potentiating their pro-tumorigenic activities [48,49].

The accumulation of lactic acid in the TME shifts macrophages towards an immune-tolerant phenotype, increasing their immunosuppressive properties; lactic acid also inhibits the production of the immunostimulatory cytokine IFNγ in T cells and NK cells [43,48,50]. 

The amino acid glutamine controls important cellular events, and recent findings demonstrated that glutamine may act as a signaling molecule, suppressing the inflammatory response in some cells, for instance adipocytes and brain microglia. TAMs express high levels of glutamine synthetase, the enzyme that synthesizes glutamine from glutamate. Recent studies have shown that increased glutamine is involved in immunosuppressive macrophage polarization and inhibition of T cell recruitment into tumors. In preclinical mouse tumor models, inhibition of glutamine synthetase alleviated immunosuppression and enhanced antitumor activity of T cells [51,52].

Other enzymes involved in the altered metabolism of tumors are well known. Arginase 1 (ARG1) and indoleamine 2,3-dioxygenase 1 (IDO1) catalyze the degradation of arginine and tryptophan, which are essential for the activation and function of T cells. The absence of these amino acids results in lower T cell survival, cytokine production and proliferation. Furthermore, tryptophan catabolism produces kynurenine, which enhances the generation of Treg cells [51].

Nucleotide metabolism also plays a crucial role in dysfunctional tumor tissues. The above-mentioned molecule adenosine, generated by tumor cells, promotes the immunosuppressive function of TAMs and MDSCs, thereby restraining the antitumor activity of cytotoxic effectors and facilitating immune evasion of cancer cells [53,54]. A recent study evaluated the role of uridine; the enzyme cytidine deaminase contributes to the uridine diphosphate (UDP) pool. Excessive extracellular UDP increases immunosuppressive TAMs; inhibition of this pathway successfully restored cytotoxic T cells and improved response to immunotherapy [52]. 

In addition to altered availability of amino acids or nucleotides, dysregulation of lipid metabolism in the tumor tissue and the TME has also received great attention, especially in the last decade. The lipid metabolic profile of macrophages can alter their functional state in a cancer-relevant manner. For example, a subpopulation of TAMs with abundant lipid content has been identified in various cancer types and has been associated with an immunosuppressive phenotype supporting tumor progression [55,56,57].

Increased lipid uptake by TAMs is mediated by lipid-scavenging receptors, in particular CD36, macrophage receptor with collagenous structure (MARCO) and TREM2. A recent study in human prostatic tumors identified a cluster of TAMs expressing the scavenger receptor MARCO and characterized by dysregulated lipid metabolism; these lipid-loaded TAMs are associated with shorter disease-free survival in patients [58]. 

Overall, the dysfunctional metabolism present in the tumor tissue is complex; alterations of specific metabolic products, enzymes, nucleotides and amino acids, as well as lipids, contribute to shifting the functional activities of tumor-infiltrating leukocytes towards a state of immunosuppression, thus promoting immune evasion of cancer cells. 

## 5. Endoplasmic Reticulum Stress

Tumor cells require extensive protein synthesis and folding in the endoplasmic reticulum (ER) to maintain their continuous replication. This increased demand, together with other cellular stressors described above, such as genomic instability, nutrient deprivation, oxidative stress and hypoxia, can lead to perturbation of ER homeostasis. The accumulation of misfolded proteins in the ER activates the unfolded protein response (UPR), a stress response that, by arresting protein translation and increasing molecular chaperones involved in protein folding, aims to restore normal functions. If the damage is irreversible, the UPR ultimately leads to cell apoptosis. However, tumor cells have developed mechanisms to use ER stress to their advantage to continue proliferating [59,60,61,62,63,64]. Activation of the UPR involves the action of three ER transmembrane sensors: inositol-requiring enzyme 1α (IRE1α), protein kinase RNA-like ER kinase (PERK) and activating transcription factor 6 (ATF6). In homeostatic conditions, these sensors are inert by association with the chaperone GRP78, a member of the Hsp70 family, while dissociating and becoming activated under stress conditions. GRP94, another chaperone of the Hsp90 family, is also specifically upregulated by a variety of stress conditions that perturb ER functions [59,65,66].

In cancer cells, activation of IRE1α in combination with HIF1α promotes resistance to hypoxia; it also regulates cellular metabolism by expressing GLUT1 and LDHA, allowing tumor cells to consume glucose and enhance glycolysis. PERK and ATF6 signaling suppress caspase-mediated apoptosis and enable tumor cell survival. Therefore, prolonged ER stress can increase cancer cell proliferation and metastatic capacity, promote neo-angiogenesis and drug resistance, and produce immunosuppressive mediators [59,60].

The ER-stressed tumor environment also has a heavy impact on other local cell populations. Macrophages and lymphocytes are known to exhibit irregular UPR in the endoplasmic reticulum and dysfunctional mitochondria. These conditions profoundly affect their efficiency as antitumor effectors [67]. In macrophages, IRE1α influences their functional polarization, promoting immune-tolerant differentiation. PERK activation in TAMs enhances mitochondrial function and epigenetic reprogramming, leading to increased immunosuppressive function [68]. 

In T lymphocytes, IRE1α perturbs N-linked glycosylation and mitochondrial respiration, resulting in decreased IFNγ production and increased expression of immune checkpoints and exhaustion markers. Activation of PERK expression in T cells is associated with inhibition of T-bet, the master transcription factor mediating Th1 differentiation, as well as increased ROS production [63,69].

Of note, exposure of immune cells to soluble factors derived from ER-stressed tumors can induce an intrinsic UPR condition, raising the concept of a transmissible ER stress response [70]. 

Tumor-derived lipids or proteins, in the form of microvesicles or soluble material, can modify the homeostasis of other local cells. For instance, membrane phospholipid alteration causes chronic ER stress in other cells; tumor lipids can induce IRE1α in myeloid cells, excess lipid accumulation and impaired Ag presentation. Activation of UPR can inhibit the surface expression of MHC-I molecules, an effect that contributes to defective Antigen Presenting Cell (APC) function [71,72,73]. 

In summary, activation of the UPR stress in tumor-infiltrating immune cells affects a variety of molecular pathways, overall leading to functional differentiation of tolerogenic or exhausted cells and to increased survival of immunosuppressive cells.

## 6. Opportunities for Therapeutic Intervention

Understanding cellular stress mechanisms in the tumor tissue and their involvement in resistance to therapeutic treatments is of great importance. Numerous pathways related to cellular stress have been considered in recent years as potential targets of specific pharmacological interventions, also directed against immune cells in the microenvironment. Identifying the best targeting strategies and using them in treatments, in combination with conventional therapies, can offer more effective treatments for patients (Table 1).

### 6.1. Hypoxia

Hypoxia creates a favorable environment for tumor progression by shaping the landscape of the TME that promotes angiogenesis, metabolic adaptation, immune escape and metastasis. Numerous HIF inhibitors have been synthesized and studied. The HIF-1α inhibitor PX-478 has been studied in several murine tumor models. Its administration alone had no antitumor effect, but combining it with chemotherapy or checkpoint blockade immunotherapy improved the response and resulted in greater inhibition of tumor growth compared to single treatments. An effect was also observed in immune cells: an increased infiltration of CD8 T cells within the tumor was detected, as well as a decrease in immunosuppressive TAMs and MDSCs. Other inhibitors of HIF1α or HIF2 have been tested in vitro and in vivo in preclinical models with similar results [27,74,75]. More advanced studies are available using the selective HIF-2 inhibitor belzutifan, which was approved by the FDA in 2021 for the treatment of clear cell renal cell carcinoma. A substantial proportion of these renal tumors have mutations in the *Von Hippel–Lindau* (*VHL*) tumor suppressor gene, a master regulator of the HIF signaling pathway. Loss of *VHL* function leads to accumulation of HIF2α and increased probability of malignant transformation. Clinical trials with belzutifan monotherapy have improved the clinical outcome in some patients. Combination studies with other compounds: tyrosine kinase inhibitors or anti-checkpoint antibodies are currently ongoing, including some more advanced phase 3 clinical trials in renal cancer or other *VHL*-associated cancers [27,75,78]. 

Another approach is to use compounds that are activated under hypoxic conditions. Hypoxia-activated prodrugs (HAPs) become cytotoxic only in hypoxic regions of tumor tissue, almost sparing normoxic areas. Several HAPs have undergone preclinical studies. Evofosfamide (TH-302) has been shown to sensitize tumors to checkpoint immunotherapy and enhance T cell infiltration in various mouse cancer models. Evofosfamide is currently undergoing phase II and III clinical evaluation in combination with chemotherapy or immunotherapy [76,77,79].

### 6.2. Oxidative Stress

Cellular redox potential is an important mechanism for balancing the harmful effects of reactive oxygen and nitrogen species. Cells have developed natural antioxidant systems to defend themselves from oxidative stress. The enzymes glutathione peroxidase, thioredoxin, catalase and superoxide dismutase remove H_2_O_2_ and superoxide anions in an attempt to neutralize the oxidative stress. Other non-enzymatic compounds with antioxidant activity are, for example, vitamin C, vitamin E, carotenoids, flavonoids and elements such as selenium and zinc. These antioxidants are present in foods and are usually taken with a balanced diet or as food supplements. These compounds or nutritional elements are powerful antioxidants and are of major importance for our health. Their potential anti-tumor effects, especially high-dose vitamin E or vitamin C, have been tested as pharmacological approaches in mouse cancer models, overall without satisfactory results. Their contribution in improving the response to checkpoint inhibitors has also been the object of investigation [39,80,81,82,83,84].

As mentioned above, the role of the transcription factor NRF2 in cancer is controversial. Some studies suggest that NRF2 may act as a tumor suppressor, inhibiting carcinogenesis. However, NRF2 expression is elevated in many types of tumors and is associated with poor prognosis, as it provides cancer cells with a survival advantage and resistance to therapies [84,85].

In principle, NRF2 activators could be used for the prevention of chemical carcinogenesis, while NRF2 inhibitors could be used for the treatment of already established cancers or to sensitize tumor cells to other therapies. In any case, the development of therapeutic strategies based on NRF2 modulation requires meticulous evaluation. Some NRF2 inhibitors are being evaluated in clinical trials, but so far with limited success due to two main problems: limited antitumor activity and significant toxicity [86].

Since NRF2 activity is also elevated in myeloid cells present in the TME, future studies to identify potential target molecules of NRF2 signaling in macrophages are of particular significance. Reprogramming TAMs by balancing their redox potential has a strong scientific rationale and may limit their immunosuppressive activity [46].

### 6.3. Metabolic Stress

The extreme metabolic conditions generated in the TME in terms of hypoxia and nutrient deprivation are causatively linked to the functional deterioration of infiltrating immune cells, in particular of myeloid cells. More frequently, macrophages and MDSCs are sustained and further shifted towards an immunosuppressive polarization, with a consequent negative impact on the adaptive immunity. High glycolytic activity and dysregulated lipid and amino acid metabolism, are considered potential targets of pharmacological intervention for cancer therapy [7,112].

Lactic acid accumulation and immunosuppressive polarization of TAMs can be counteracted by strategies targeting the glycolysis pathway. Numerous glycolytic enzymes or transporter molecules, such as GLUT1, have been identified as potential therapeutic targets, and various small-molecule inhibitors have been developed [87,113,114].

Although these targeting strategies have been shown to slow tumor progression and decrease the resistance to anticancer drugs, these inhibitors are far from specific and likely affect glucose metabolism even in normal tissues. Furthermore, the glycolytic pathway is particularly critical for macrophage activity against tumor cells, and glucose supply is necessary for ROS production and cell phagocytosis [7,57]. 

Metformin, a hypoglycemic agent commonly used in type 2 diabetic patients, is a pleiotropic drug with multiple effects on cellular metabolism, including inhibition of the mitochondrial respiratory chain (complex I), decreased gluconeogenesis, regulation of the AMPK pathway/mTOR implicated in the control of protein synthesis, cell proliferation, and more [87,88].

In addition to its direct effects on tumor cells, metformin has been studied for its influence on the tumor immune environment. A recent article showed the transcriptomic profile of pancreatic tumors in patients treated with metformin; the drug appears to shift the immunosuppressive TME towards an immunoactive state with antitumor properties. Metformin reduced the infiltration of pro-tumor macrophages and enhanced the recruitment of immunoactivating dendritic cells. Therefore, metformin can complement conventional anticancer treatments through its effect on cellular metabolic pathways [89,90,97,98].

Other therapeutic interventions related to macrophage metabolism have been explored in experimental tumor models in vivo. For instance, blocking glutamine metabolism by small molecules or inhibiting its synthase resulted in decreased tumor growth via modulation of suppressive myeloid cells [91,92].

Adenosine pathway inhibitors have been extensively studied. Drugs have been developed that target the cell surface ectonucleotidases CD39 and CD73 (which catalyze the generation of adenosine) or the adenosine A2A and A2B receptors on tumor-infiltrating immune cells. Results in preclinical models have been positive, and some of these drugs are being tested in clinical trials, raising hopes of counteracting tumor progression [93,94].

Inhibitors of IDO initially raised many expectations as this enzyme crucially consumes the amino acid tryptophan and severely impacts the functional status of T cells. IDO-selective therapeutic strategies have been extensively studied. Over the years, some IDO inhibitors reached the testing stage in clinical trials, also in combination with immunotherapy; however, the clinical results in phase 3 trials were mostly disappointing [99,100]. Other enzymes, in addition to IDO, are potentially involved in tryptophan catabolism, and the blockade of these enzymes in tumors is currently under way [95]. 

As mentioned above, dysfunctional lipid metabolism occurs in TAMs, and defective lipid handling results in lipid-loaded macrophages with an immunosuppressive functional phenotype. Reprogramming of TAMs by reducing their lipid uptake through scavenger receptors is a possible strategy [94]. Blockade of MARCO or CD36 significantly reduced the lipid content in TAMs and promoted tumor inhibition in preclinical models [96].

### 6.4. ER Stress

Activation of the unfolded protein response promotes tumor cell proliferation and invasion and contributes to resistance to available therapies. The relevance of these findings is confirmed by the fact that, in different studies, the genetic signatures derived from the ER stress response were found to be significantly associated with poor patient prognosis [25,60,65].

Furthermore, the UPR can be triggered also in tumor-infiltrating immune cells or “transmitted” to neighboring immune cells, thus resulting in increased immunosuppression. Therefore, these molecular pathways represent potential therapeutic targets [65,101,102]. 

One approach is to accelerate tumor cell apoptosis using proteasome inhibitors. The underlying hypothesis is that inhibition of the proteasome machinery creates an accumulation of proteins that causes faster cell death. The proteasome inhibitor bortezomib, approved by the FDA, is currently used to treat multiple myeloma. Studies have proposed that sensitivity to bortezomib is associated with levels of UPR mediators, particularly high levels of XBP-1 mRNA, generated by the endoribonuclease IRE1 [103].

In more recent years, considerable effort has been made to develop small-molecule inhibitors targeting the UPR sensors, particularly PERK1 and IRE1α, with fewer studies on the ATF6α pathway. Numerous inhibitors of PERK or IRE1α have been synthesized and tested in vitro and in preclinical in vivo models. Some of these compounds have demonstrated good antitumor effects with increased tumor cell apoptosis; effects on the immunosuppressive cells have also been reported in some studies [61,65].

Most small-molecule antagonists of IRE1α target the catalytic activity of the RNase domain or ATP-binding pocket of IRE1α. The IRE1α inhibitor STF-083010 has been shown to suppress IRE1α signaling in T cells and reprogram cholesterol metabolism in intratumoral CD8+ T cells to promote antitumor activity in murine melanoma models [104]. STF-083010 and other IRE1α inhibitors effectively reduced tumor progression in mouse models of multiple myeloma, prostate cancer and lymphoma [105,115,116]. The IRE1α inhibitor ORIN1001 is currently in clinical development in a Phase 1/2 clinical trial in patients with advanced solid tumors and metastatic breast cancer (NCT03950570). The drug sunitinib, a tyrosine kinase inhibitor approved for patients with renal cell carcinoma, also antagonizes the activity of the IRE1α kinase domain, but its role in animal models of ER stress is unclear. The small molecule KIRA8 disrupts IRE1α-dependent growth of multiple myeloma cells in mice, and it has greater efficacy in combination with the proteasome inhibitor bortezomib or the immunomodulatory agent lenalidomide [106].

For the other UPR sensor, PERK, compounds such as the small molecules GSK2606414 and GSK2656157 have been shown to inhibit its phosphorylation and to decrease tumor progression in animal models [72,107]. Administration of GSK2606414 or of another PERK inhibitor (AMG-44) also attenuated the immunosuppressive properties of MDSCs and TAMs, resulting in increased CD8+ T cell infiltration of tumor tissues and improved efficacy of anti-PD1 checkpoint therapy in mouse models [68,108,109]. Two other PERK inhibitors, HC-5404-FU and NMS-03597812, are now undergoing early clinical trials for the treatment of solid tumors (NCT04834778, NCT05027594). 

GRP78 and GRP94 are molecular chaperones of the UPR; small-molecule inhibitors have been developed, particularly against GRP94. As cell surface expression of GRP94 was demonstrated, mouse and human monoclonal antibodies have been generated for easier targeting. These compounds or antibodies are currently being evaluated for their potential anti-tumor activity. Interestingly, since GRP94 specifically interacts with HER2 on the plasma membrane of some tumor cells, blocking this interaction with specific antibodies could be of benefit in HER2-positive breast cancer [117,118]. GRP94 was found expressed at the membrane of in vitro differentiated M2 but not M1 macrophages and in CD206+ TAMs in a murine model of breast cancer. Treatment of tumor-bearing mice with PU-WS13, a GRP94 inhibitor, resulted in a significant reduction in the number of tumor-infiltrating macrophages [110]. 

GRP94 is a chaperone for various proteins, including integrins, such as LFA-1 (CD11a). A recent study reported that the genetic and pharmacological targeting of the GRP94/LFA-1 axis strongly reduced the infiltration of Tregs in the TME, potentiating the response of CD8+ T cell effectors against the tumor [111].

## 7. Conclusions

Overall, great progress has been made over the past decade in understanding the mechanisms of cellular stress in tumor tissue and their impact on disease progression. The TME, where the tumor grows and where normal stromal cells reside, primarily immune cells, expresses hostile conditions that are at the limit of cell survival, such as nutrient deprivation, oxygen limitation, oxidative stress and activation of the UPR. In general, these are mechanisms that allow tumor cells to survive, while the impact on immune cells is most commonly to have a severe functional deficit or a shift towards a suppressive phenotype. Targeting these pathways could be a promising option to reactivate immune cells and sensitize tumor cells to cytotoxic therapies and/or to immune-mediated elimination. Different cell stress pathways have been considered; several small molecule inhibitors, or blocking mAbs, have been generated, screened and selected in preclinical mouse tumor models and early clinical trials. Some of these studies have indicated that inhibition of cellular stress pathways has promising applications for future therapies. The identification of the most promising approaches, and especially the best combination with existing therapies, is expected to have a significant impact in the development of new treatments for cancer patients.

## Figures and Tables

**Table 1:** Candidate and approved therapeutic compounds targeting stress pathways in the TME.

	Categories/Compounds	Preclinical Studies	Clinical Studies
Hypoxic Stress	HIF1a-HIF2 inhibitors Hypoxia-activated Pro-drug	Px-478 Other [27,74,75] Evofosfamide TH-302 [76,77]	Belzutifan (FDA approved) [25,75,78] Evofosfamide (phase II and III) [76,77,79]
Oxidative Stress	Antioxidants (diet and supplements) NRF2 inhibitors	Vit. C, Vit. E [80,81,82,83] [84,85,86]	(phase I/II and III) (phase I and II) [84,86]
Metabolic Stress	Hypoglycemic agents Blockers glutamine metabolism Blockers adenosine metabolism IDO inhibitors Blockers of scavenger receptors	Metformin [87,88,89,90] [91,92] [93,94] [95] [94,96]	Metformin (FDA approved) [97,98] NA NA (phase III) [95,99,100] NA
Endoplasmic Reticulum stress	Proteome Inhibitors Inhibitors of PERK1, IRE1a, ATF6a GRP94 inhibitors	Bortezomib [65,101,102,103] [61,65,68,72,104,105,106,107,108,109] [110,111]	Bortezomib (FDA approved) [101,103] PERK inhibitors (Phase I-II) IRE1a inhibitors (Phase I-II) NA

NA: not applicable.
